# (*E*)-Methyl 2-chloro-4-dicyclo­hexyl­amino-4-oxobut-2-enoate

**DOI:** 10.1107/S1600536811027760

**Published:** 2011-07-16

**Authors:** Cai-Mei Liu, Fu-Ling Xue, Jian-Hua Fu, Zhao-Yang Wang

**Affiliations:** a School of Chemistry and Environment, South China Normal University, Guangzhou 510006, People’s Republic of China

## Abstract

In the title compound, C_17_H_26_ClNO_3_, both cyclo­hexyl rings have chair conformations. In the crystal, mol­ecules are linked by weak inter­molecular C—H⋯O hydrogen bonds.

## Related literature

For the synthesis, see: Song *et al.* (2009 ▶). For the biological activity of 2(5*H*)-furan­ones, see: Lattmann *et al.* (2005 ▶); Rowland *et al.* (2007 ▶); Kim *et al.* (2002 ▶). For chemical, pharmaceutical and agrochemical applications of 3,4-amino-2(5*H*)-furanones, see: Kimura *et al.* (2000 ▶); Tanoury *et al.* (2008 ▶). For related structures, see: Lattmann *et al.* (1999 ▶, 2006 ▶).
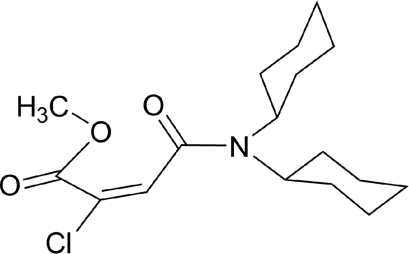


## Experimental

### 

#### Crystal data

C_17_H_26_ClNO_3_
*M* *_r_* = 327.84
Monoclinic, 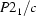

*a* = 8.8291 (19) Å
*b* = 10.533 (2) Å
*c* = 19.139 (4) Å
β = 92.955 (3)°
*V* = 1777.5 (6) Å^3^
*Z* = 4
Mo *K*α radiation
μ = 0.23 mm^−1^
*T* = 298 K
0.32 × 0.22 × 0.20 mm

#### Data collection

Bruker APEXII area-detector diffractometer
Absorption correction: multi-scan (*SADABS*; Sheldrick, 1996 ▶) *T*_min_ = 0.931, *T*_max_ = 0.956
8635 measured reflections
3886 independent reflections
2457 reflections with *I* > 2σ(*I*)
*R*_int_ = 0.051

#### Refinement

*R*[*F* ^2^ > 2σ(*F* ^2^)] = 0.053
*wR*(*F* ^2^) = 0.160
*S* = 1.02
3886 reflections
201 parameters
H-atom parameters constrained
Δρ_max_ = 0.24 e Å^−3^
Δρ_min_ = −0.28 e Å^−3^

### 

Data collection: *APEX2* (Bruker, 2008 ▶); cell refinement: *SAINT* (Bruker, 2008 ▶); data reduction: *SAINT*; program(s) used to solve structure: *SHELXS97* (Sheldrick, 2008 ▶); program(s) used to refine structure: *SHELXL97* (Sheldrick, 2008 ▶); molecular graphics: *ORTEP-3 for Windows* (Farrugia, 1997 ▶); software used to prepare material for publication: *SHELXL97*.

## Supplementary Material

Crystal structure: contains datablock(s) global, I. DOI: 10.1107/S1600536811027760/lx2191sup1.cif

Structure factors: contains datablock(s) I. DOI: 10.1107/S1600536811027760/lx2191Isup2.hkl

Supplementary material file. DOI: 10.1107/S1600536811027760/lx2191Isup3.cml

Additional supplementary materials:  crystallographic information; 3D view; checkCIF report

## Figures and Tables

**Table 1:** Hydrogen-bond geometry (Å, °)

*D*—H⋯*A*	*D*—H	H⋯*A*	*D*⋯*A*	*D*—H⋯*A*
C17—H17*A*⋯O1^i^	0.96	2.44	3.323 (4)	153
C9—H9⋯O2^ii^	0.93	2.50	3.389 (3)	160

Symmetry codes: (i) 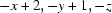; (ii) 
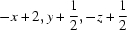
.

**Fig. 1.**
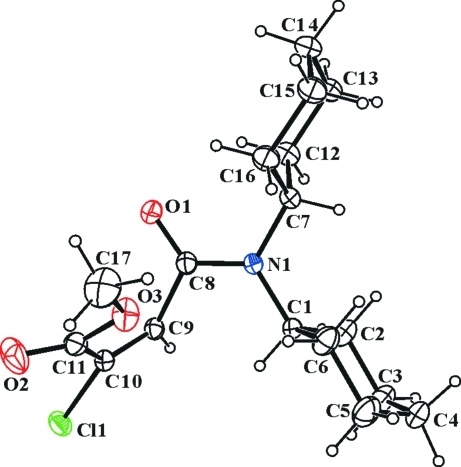
The molecular structure of the title compound showing the atom-labelling scheme. Ellipsoids are drawn at the 50% probability level. H atoms are presented as a small spheres of arbitrary radius.

**Fig. 2.**
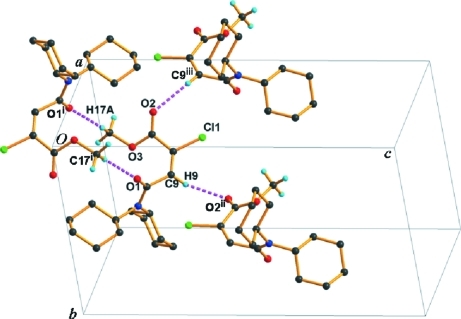
A view of the C—H···O hydrogen bonds (dotted lines) in the crystal structure of the title compound. [Symmetry codes: (i) - x + 2, - y + 1, - z; (ii) - x + 2, y + 1/2, - z + 1/2; (iii) - x + 2, y - 1/2, - z + 1/2.]

## supplementary crystallographic information

### Comment

Molecules possessing 2(5H)-furanone moiety are useful heterocyclic compounds due to their valuable biological activities such as antibacterial, anti-inflammatory and antitumor (Lattmann *et al.*, 2005; Rowland *et al.*, 2007; Kim *et al.*, 2002). The 5-alkoxy-3,4-dihalo-2(5H)-furanones being a kind of synthons are widely used in tandem Michael addition-elimination reactions (Song *et al.*, 2009). The 4-amino-2(5H)-furanones exhibit an antibiotic activity against MRSA (Lattmann *et al.*, 1999; Lattmann *et al.*, 2006). Therefore, we are interested in the tandem Michael addition-elimination reaction of the synthon 3,4-dichloro-5-methoxyfuran-2(5H)-one with secondary amines in the present of potassium fluoride. However, we obtained an unanticipated product, (E)-methyl 2-chloro-4-(dicyclohexylamino)-4-oxobut-2-enoate, instead of the expected compound 3-chloro-4-(dicyclohexylamino)-5-methoxyfuran-2(5H)-one (Lattmann *et al.*, 1999). Herein, we report the crystal structure of the title compound.

In the title compound (Fig. 1), the both cyclohexyl rings are in the chair form. The molecular packing (Fig. 2) is stabilized by weak intermolecular C—H···O hydrogen bonds; the first one between the H atom of the vinyl group and the O atom of the ester group (Table 1; C9—H9···O2^ii^), and a methyl H atom and the O atom of the amide group (Table 1; C17—H17A···O1^i^).

### Experimental

The precursor 3,4-dichloro-5-methoxyfuran-2(5H)-one was prepared according to the literature procedure (Song *et al.*, 2009). The solution of dicyclohexylamine (2.736 g, 3.0 mL) in tetrahydrofuran (3.0 mL) was added to a stirred solution of 3,4-dichloro-5-methoxyfuran-(5H)-one (36.39 mg, 2.0 mmol) and potassium fluoride (34.85 mg, 6.0 mmol) in tetrahydrofuran (3.0 mL) under nitrogen atmosphere. After being stirred at room temperature for 24 h, the solvent was removed under reduced pressure. The residual solid was dissolved in dichloromethane and then the combined organic layer was concentrated under reduced pressure. The residue was purified by silica gel column chromatography with the gradient mixture of petroleum ether and ethyl acetate to give the title compound (yield 22.6%). Single crystals suitable for X-ray diffraction were prepared by slow evaporation of a solution of the title compound in (insert proper solvent) at room temperature.

### Refinement

H atoms were positioned in calculated positions with C—H = 0.93–0.98 Å and were refined using a riding model, with *U*_iso_(H) = 1.5*U*_eq_(C) for methyl and 1.2*U*_eq_(C) for the others.

### Crystal data

**Table:**

C_17_H_26_ClNO_3_	*F*(000) = 704
*M**_r_* = 327.84	*D*_x_ = 1.225 Mg m^−^^3^
Monoclinic, *P*2_1_/*c*	Mo *K*α radiation, λ = 0.71073 Å
Hall symbol: -P 2ybc	Cell parameters from 2253 reflections
*a* = 8.8291 (19) Å	θ = 2.2–23.5°
*b* = 10.533 (2) Å	µ = 0.23 mm^−^^1^
*c* = 19.139 (4) Å	*T* = 298 K
β = 92.955 (3)°	Block, colourless
*V* = 1777.5 (6) Å^3^	0.32 × 0.22 × 0.20 mm
*Z* = 4	

### Data collection

**Table:**

Bruker APEXII area-detector diffractometer	3886 independent reflections
Radiation source: fine-focus sealed tube	2457 reflections with *I* > 2σ(*I*)
graphite	*R*_int_ = 0.051
Detector resolution: 10.0 pixels mm^-1^	θ_max_ = 27.0°, θ_min_ = 2.1°
φ and ω scans	*h* = −4→11
Absorption correction: multi-scan (*SADABS*; Sheldrick, 1996)	*k* = −13→12
*T*_min_ = 0.931, *T*_max_ = 0.956	*l* = −24→24
8635 measured reflections	

### Refinement

**Table:**

Refinement on *F*^2^	Secondary atom site location: difference Fourier map
Least-squares matrix: full	Hydrogen site location: difference Fourier map
*R*[*F*^2^ > 2σ(*F*^2^)] = 0.053	H-atom parameters constrained
*wR*(*F*^2^) = 0.160	*w* = 1/[σ^2^(*F*_o_^2^) + (0.070*P*)^2^] where *P* = (*F*_o_^2^ + 2*F*_c_^2^)/3
*S* = 1.02	(Δ/σ)_max_ = 0.001
3886 reflections	Δρ_max_ = 0.24 e Å^−^^3^
201 parameters	Δρ_min_ = −0.28 e Å^−^^3^
0 restraints	Extinction correction: *SHELXL97* (Sheldrick, 2008), Fc^*^=kFc[1+0.001xFc^2^λ^3^/sin(2θ)]^-1/4^
Primary atom site location: structure-invariant direct methods	Extinction coefficient: 0.015 (3)

### Special details

**Table:**

Experimental. Data for (I): ^1^H NMR (400 MHz, CDCl_3_, TMS): 1.073-1.564 (12H, *m*, 6CH_2_), 1.681-2.436 (8H, *m*, 4CH_2_), 2.973-3.124 (1H, *m*, CH), 3.350-3.417 (1H, *m*, CH), 3.813 (3H, *s*, CH_3_), ESI-MS, *m*/*z* (%): Calcd for C_17_H_27_ClNO_3_^+^([M+H]^+^): 328.16(100.0), 330.16(32.6), found: 328.39(15.0), 330.43(5.0).
Geometry. All esds (except the esd in the dihedral angle between two l.s. planes) are estimated using the full covariance matrix. The cell esds are taken into account individually in the estimation of esds in distances, angles and torsion angles; correlations between esds in cell parameters are only used when they are defined by crystal symmetry. An approximate (isotropic) treatment of cell esds is used for estimating esds involving l.s. planes.
Refinement. Refinement of F^2^ against ALL reflections. The weighted R-factor wR and goodness of fit S are based on F^2^, conventional R-factors R are based on F, with F set to zero for negative F^2^. The threshold expression of F^2^ > 2sigma(F^2^) is used only for calculating R-factors(gt) etc. and is not relevant to the choice of reflections for refinement. R-factors based on F^2^ are statistically about twice as large as those based on F, and R- factors based on ALL data will be even larger.

### Fractional atomic coordinates and isotropic or equivalent isotropic displacement parameters (Å^2^)

**Table:**

	*x*	*y*	*z*	*U*_iso_*/*U*_eq_	
Cl1	1.02330 (7)	0.47301 (6)	0.29004 (3)	0.0574 (2)	
N1	0.7284 (2)	0.73071 (19)	0.10932 (8)	0.0427 (5)	
C1	0.6049 (2)	0.7104 (2)	0.15716 (10)	0.0452 (6)	
H1	0.6483	0.6628	0.1974	0.054*	
C6	0.4768 (3)	0.6305 (3)	0.12475 (14)	0.0623 (7)	
H6A	0.4338	0.6728	0.0833	0.075*	
H6B	0.5167	0.5492	0.1105	0.075*	
C3	0.4221 (3)	0.8114 (3)	0.23589 (14)	0.0711 (8)	
H3A	0.4656	0.7696	0.2774	0.085*	
H3B	0.3815	0.8924	0.2501	0.085*	
C2	0.5460 (3)	0.8343 (3)	0.18519 (12)	0.0588 (7)	
H2A	0.5062	0.8861	0.1465	0.071*	
H2B	0.6288	0.8804	0.2088	0.071*	
C4	0.2953 (3)	0.7311 (3)	0.20438 (14)	0.0662 (8)	
H4A	0.2238	0.7126	0.2398	0.079*	
H4B	0.2421	0.7781	0.1671	0.079*	
C5	0.3537 (3)	0.6090 (3)	0.17577 (18)	0.0805 (9)	
H5A	0.2704	0.5633	0.1523	0.097*	
H5B	0.3937	0.5568	0.2142	0.097*	
C9	0.9064 (2)	0.6357 (2)	0.19601 (10)	0.0407 (5)	
H9	0.9020	0.6907	0.2338	0.049*	
C10	0.9445 (2)	0.5170 (2)	0.20899 (10)	0.0414 (5)	
C8	0.8696 (2)	0.6886 (2)	0.12389 (10)	0.0431 (5)	
C7	0.6909 (3)	0.7947 (2)	0.04169 (10)	0.0469 (6)	
H7	0.5843	0.8202	0.0426	0.056*	
C11	0.9352 (3)	0.4105 (2)	0.15775 (11)	0.0519 (6)	
C12	0.7807 (3)	0.9153 (3)	0.03266 (13)	0.0625 (7)	
H12A	0.8878	0.8952	0.0320	0.075*	
H12B	0.7662	0.9716	0.0718	0.075*	
C16	0.7013 (3)	0.7045 (3)	−0.02024 (11)	0.0617 (7)	
H16A	0.6369	0.6312	−0.0140	0.074*	
H16B	0.8049	0.6752	−0.0233	0.074*	
C13	0.7293 (4)	0.9818 (3)	−0.03560 (14)	0.0788 (10)	
H13A	0.6254	1.0104	−0.0327	0.095*	
H13B	0.7924	1.0557	−0.0423	0.095*	
C15	0.6504 (4)	0.7749 (4)	−0.08734 (13)	0.0845 (11)	
H15A	0.6612	0.7190	−0.1271	0.101*	
H15B	0.5439	0.7967	−0.0855	0.101*	
C14	0.7399 (4)	0.8930 (4)	−0.09763 (14)	0.0881 (12)	
H14A	0.7018	0.9357	−0.1399	0.106*	
H14B	0.8452	0.8710	−0.1033	0.106*	
O1	0.97348 (18)	0.69654 (19)	0.08431 (8)	0.0573 (5)	
O3	0.8279 (2)	0.43037 (19)	0.10887 (9)	0.0648 (5)	
O2	1.0144 (3)	0.3193 (2)	0.16138 (10)	0.0985 (9)	
C17	0.8142 (4)	0.3387 (4)	0.05276 (16)	0.0938 (11)	
H17A	0.8939	0.3520	0.0213	0.141*	
H17B	0.7177	0.3487	0.0279	0.141*	
H17C	0.8218	0.2545	0.0718	0.141*	

### Atomic displacement parameters (Å^2^)

**Table:**

	*U*^11^	*U*^22^	*U*^33^	*U*^12^	*U*^13^	*U*^23^
Cl1	0.0631 (4)	0.0582 (4)	0.0499 (3)	0.0121 (3)	−0.0066 (3)	0.0093 (2)
N1	0.0360 (10)	0.0521 (12)	0.0399 (9)	0.0072 (8)	0.0010 (7)	0.0075 (7)
C1	0.0338 (11)	0.0604 (16)	0.0417 (11)	0.0065 (10)	0.0025 (8)	0.0121 (10)
C6	0.0532 (15)	0.0544 (18)	0.0806 (17)	−0.0074 (12)	0.0148 (13)	−0.0088 (13)
C3	0.0494 (15)	0.105 (3)	0.0594 (14)	−0.0008 (15)	0.0108 (12)	−0.0137 (15)
C2	0.0438 (13)	0.0717 (19)	0.0617 (14)	−0.0059 (12)	0.0110 (11)	−0.0175 (12)
C4	0.0413 (14)	0.084 (2)	0.0744 (17)	0.0032 (13)	0.0129 (12)	0.0066 (14)
C5	0.0538 (17)	0.071 (2)	0.118 (2)	−0.0079 (15)	0.0236 (16)	0.0114 (17)
C9	0.0358 (11)	0.0439 (14)	0.0422 (10)	0.0024 (9)	−0.0014 (8)	−0.0004 (9)
C10	0.0362 (11)	0.0462 (14)	0.0417 (10)	0.0056 (9)	0.0023 (8)	0.0038 (9)
C8	0.0410 (12)	0.0448 (14)	0.0434 (11)	0.0036 (10)	−0.0004 (9)	−0.0023 (9)
C7	0.0440 (12)	0.0558 (15)	0.0408 (10)	0.0090 (11)	0.0004 (9)	0.0082 (9)
C11	0.0612 (15)	0.0469 (15)	0.0486 (12)	0.0088 (12)	0.0128 (11)	0.0018 (10)
C12	0.0655 (17)	0.0614 (19)	0.0607 (14)	0.0049 (14)	0.0044 (12)	0.0136 (12)
C16	0.0622 (16)	0.078 (2)	0.0440 (12)	0.0043 (14)	−0.0025 (11)	−0.0009 (11)
C13	0.081 (2)	0.079 (2)	0.0781 (19)	0.0246 (17)	0.0235 (16)	0.0392 (16)
C15	0.080 (2)	0.129 (3)	0.0432 (13)	0.022 (2)	−0.0034 (13)	0.0081 (15)
C14	0.092 (2)	0.119 (3)	0.0547 (15)	0.040 (2)	0.0198 (15)	0.0367 (17)
O1	0.0416 (9)	0.0774 (14)	0.0534 (9)	0.0111 (8)	0.0070 (7)	0.0084 (8)
O3	0.0602 (11)	0.0674 (13)	0.0654 (10)	0.0056 (9)	−0.0101 (9)	−0.0238 (9)
O2	0.150 (2)	0.0773 (17)	0.0678 (12)	0.0605 (16)	−0.0023 (13)	−0.0091 (10)
C17	0.107 (3)	0.096 (3)	0.0781 (19)	0.003 (2)	−0.0031 (18)	−0.0433 (18)

### Geometric parameters (Å, °)

**Table:**

Cl1—C10	1.730 (2)	C8—O1	1.222 (2)
N1—C8	1.339 (3)	C7—C12	1.512 (4)
N1—C1	1.475 (3)	C7—C16	1.525 (3)
N1—C7	1.481 (3)	C7—H7	0.9800
C1—C2	1.514 (4)	C11—O2	1.189 (3)
C1—C6	1.517 (3)	C11—O3	1.313 (3)
C1—H1	0.9800	C12—C13	1.530 (3)
C6—C5	1.515 (3)	C12—H12A	0.9700
C6—H6A	0.9700	C12—H12B	0.9700
C6—H6B	0.9700	C16—C15	1.530 (4)
C3—C4	1.504 (4)	C16—H16A	0.9700
C3—C2	1.518 (3)	C16—H16B	0.9700
C3—H3A	0.9700	C13—C14	1.518 (5)
C3—H3B	0.9700	C13—H13A	0.9700
C2—H2A	0.9700	C13—H13B	0.9700
C2—H2B	0.9700	C15—C14	1.493 (5)
C4—C5	1.500 (4)	C15—H15A	0.9700
C4—H4A	0.9700	C15—H15B	0.9700
C4—H4B	0.9700	C14—H14A	0.9700
C5—H5A	0.9700	C14—H14B	0.9700
C5—H5B	0.9700	O3—C17	1.444 (3)
C9—C10	1.315 (3)	C17—H17A	0.9600
C9—C8	1.508 (3)	C17—H17B	0.9600
C9—H9	0.9300	C17—H17C	0.9600
C10—C11	1.489 (3)		
			
C8—N1—C1	122.19 (17)	N1—C8—C9	117.89 (18)
C8—N1—C7	119.78 (17)	N1—C7—C12	112.77 (19)
C1—N1—C7	117.97 (16)	N1—C7—C16	112.1 (2)
N1—C1—C2	111.9 (2)	C12—C7—C16	112.4 (2)
N1—C1—C6	112.71 (18)	N1—C7—H7	106.3
C2—C1—C6	111.2 (2)	C12—C7—H7	106.3
N1—C1—H1	106.9	C16—C7—H7	106.3
C2—C1—H1	106.9	O2—C11—O3	124.8 (2)
C6—C1—H1	106.9	O2—C11—C10	123.9 (2)
C5—C6—C1	111.3 (2)	O3—C11—C10	111.3 (2)
C5—C6—H6A	109.4	C7—C12—C13	110.4 (2)
C1—C6—H6A	109.4	C7—C12—H12A	109.6
C5—C6—H6B	109.4	C13—C12—H12A	109.6
C1—C6—H6B	109.4	C7—C12—H12B	109.6
H6A—C6—H6B	108.0	C13—C12—H12B	109.6
C4—C3—C2	112.3 (2)	H12A—C12—H12B	108.1
C4—C3—H3A	109.2	C7—C16—C15	108.9 (2)
C2—C3—H3A	109.2	C7—C16—H16A	109.9
C4—C3—H3B	109.2	C15—C16—H16A	109.9
C2—C3—H3B	109.2	C7—C16—H16B	109.9
H3A—C3—H3B	107.9	C15—C16—H16B	109.9
C1—C2—C3	111.2 (2)	H16A—C16—H16B	108.3
C1—C2—H2A	109.4	C14—C13—C12	111.0 (2)
C3—C2—H2A	109.4	C14—C13—H13A	109.4
C1—C2—H2B	109.4	C12—C13—H13A	109.4
C3—C2—H2B	109.4	C14—C13—H13B	109.4
H2A—C2—H2B	108.0	C12—C13—H13B	109.4
C5—C4—C3	111.5 (2)	H13A—C13—H13B	108.0
C5—C4—H4A	109.3	C14—C15—C16	112.3 (3)
C3—C4—H4A	109.3	C14—C15—H15A	109.1
C5—C4—H4B	109.3	C16—C15—H15A	109.1
C3—C4—H4B	109.3	C14—C15—H15B	109.1
H4A—C4—H4B	108.0	C16—C15—H15B	109.1
C4—C5—C6	112.2 (3)	H15A—C15—H15B	107.9
C4—C5—H5A	109.2	C15—C14—C13	110.9 (2)
C6—C5—H5A	109.2	C15—C14—H14A	109.5
C4—C5—H5B	109.2	C13—C14—H14A	109.5
C6—C5—H5B	109.2	C15—C14—H14B	109.5
H5A—C5—H5B	107.9	C13—C14—H14B	109.5
C10—C9—C8	124.41 (19)	H14A—C14—H14B	108.1
C10—C9—H9	117.8	C11—O3—C17	116.9 (2)
C8—C9—H9	117.8	O3—C17—H17A	109.5
C9—C10—C11	125.92 (19)	O3—C17—H17B	109.5
C9—C10—Cl1	120.73 (17)	H17A—C17—H17B	109.5
C11—C10—Cl1	113.26 (17)	O3—C17—H17C	109.5
O1—C8—N1	124.64 (19)	H17A—C17—H17C	109.5
O1—C8—C9	117.33 (19)	H17B—C17—H17C	109.5
			
C8—N1—C1—C2	116.2 (2)	C10—C9—C8—N1	115.7 (3)
C7—N1—C1—C2	−66.8 (3)	C8—N1—C7—C12	−61.9 (3)
C8—N1—C1—C6	−117.6 (2)	C1—N1—C7—C12	121.0 (2)
C7—N1—C1—C6	59.4 (3)	C8—N1—C7—C16	66.2 (3)
N1—C1—C6—C5	178.7 (2)	C1—N1—C7—C16	−110.9 (2)
C2—C1—C6—C5	−54.7 (3)	C9—C10—C11—O2	152.5 (3)
N1—C1—C2—C3	−178.38 (19)	Cl1—C10—C11—O2	−23.9 (3)
C6—C1—C2—C3	54.6 (3)	C9—C10—C11—O3	−28.0 (3)
C4—C3—C2—C1	−54.5 (3)	Cl1—C10—C11—O3	155.65 (17)
C2—C3—C4—C5	53.9 (4)	N1—C7—C12—C13	−176.0 (2)
C3—C4—C5—C6	−53.9 (3)	C16—C7—C12—C13	56.0 (3)
C1—C6—C5—C4	54.5 (3)	N1—C7—C16—C15	176.1 (2)
C8—C9—C10—C11	−10.0 (4)	C12—C7—C16—C15	−55.7 (3)
C8—C9—C10—Cl1	166.08 (16)	C7—C12—C13—C14	−55.2 (3)
C1—N1—C8—O1	175.6 (2)	C7—C16—C15—C14	56.3 (3)
C7—N1—C8—O1	−1.4 (4)	C16—C15—C14—C13	−57.4 (3)
C1—N1—C8—C9	−8.9 (3)	C12—C13—C14—C15	56.2 (4)
C7—N1—C8—C9	174.2 (2)	O2—C11—O3—C17	−5.0 (4)
C10—C9—C8—O1	−68.4 (3)	C10—C11—O3—C17	175.5 (2)

### Hydrogen-bond geometry (Å, °)

**Table:**

*D*—H···*A*	*D*—H	H···*A*	*D*···*A*	*D*—H···*A*
C17—H17A···O1^i^	0.96	2.44	3.323 (4)	153.
C9—H9···O2^ii^	0.93	2.50	3.389 (3)	160.

Symmetry codes: (i) −*x*+2, −*y*+1, −*z*; (ii) −*x*+2, *y*+1/2, −*z*+1/2.

## References

[bb1] Bruker (2008). *APEX2* and *SAINT* Bruker AXS Inc., Madison, Wisconsin, USA.

[bb2] Farrugia, L. J. (1997). *J. Appl. Cryst.* **30**, 565.

[bb3] Kim, Y., Nam, N.-H., You, Y.-J. & Ahn, B.-Z. (2002). *Bioorg. Med. Chem. Lett.* **12**, 719–722. 10.1016/s0960-894x(01)00831-9 11844709

[bb4] Kimura, Y., Mizuno, T., Kawano, T., Okada, K. & Shimad, A. (2000). *Phytochemistry*, **53**, 829–831. 10.1016/s0031-9422(99)00492-6 10820786

[bb5] Lattmann, E., Billington, D. C. & Langley, C. A. (1999). *Drug Des. Discov.* **16**, 243–250. 10624570

[bb6] Lattmann, E., Dunn, S., Niamsanit, S. & Sattayasai, N. (2005). *Bioorg. Med. Chem. Lett.* **15**, 919–921. 10.1016/j.bmcl.2004.12.051 15686887

[bb7] Lattmann, E., Sattayasai, N., Schwalbe, C. S., Niamsanit, S., Billington, D. C., Lattmann, P., Langley, C. A., Singh, H. & Dunn, S. (2006). *Curr. Drug Discov. Technol.* **3**, 125–134. 10.2174/157016306778108857 16925520

[bb8] Rowland, S., Clark, P., Gordon, R., Mullen, A., Guay, J., Dufresne, L., Brideau, C., Cote, B., Ducharme, Y. & Mancini, J. (2007). *Eur. J. Pharmacol.* **560**, 216–224. 10.1016/j.ejphar.2007.01.008 17316604

[bb9] Sheldrick, G. M. (1996). *SADABS* University of Göttingen, Germany.

[bb10] Sheldrick, G. M. (2008). *Acta Cryst.* A**64**, 112–122. 10.1107/S0108767307043930 18156677

[bb11] Song, X.-M., Wang, Z.-Y., Li, J.-X. & Fu, J.-H. (2009). *Chin. J. Org. Chem.* **11**, 1804–1810.

[bb12] Tanoury, G. J., Chen, M. Z., Dong, Y., Forslund, R. E. & Magdziak, D. (2008). *Org. Lett.* **10**, 185–188. 10.1021/ol702532h 18081302

